# Functional and Structural Determinants of Human Sperm Cryoresistance: Insights from Motility, Mitochondrial and Chromatin Integrity Analyses

**DOI:** 10.3390/cells15161480

**Published:** 2026-08-18

**Authors:** Eva Tvrdá, Temidayo S. Omolaoye, Michal Ďuračka, Fawzia AlObeidli, Stefan S. Du Plessis

**Affiliations:** 1 Institute of Biotechnology, Faculty of Biotechnology and Food Sciences, Slovak University of Agriculture in Nitra, 94976 Nitra, Slovakia; 2 Chair of Animal Breeding and Biotechnology, Institute of Veterinary Medicine and Animal Sciences, Estonian University of Life Sciences, 51008 Tartu, Estonia; 3 College of Medicine, Mohammed Bin Rashid University of Medicine and Health Sciences, Dubai Health, Dubai P.O. Box 505055, United Arab Emirates; temidayo.omolaoye@dubaihealth.ae (T.S.O.); fawzia.alobeidli@students.mbru.ac.ae (F.A.); stefan.duplessis@dubaihealth.ae (S.S.D.P.); 4 Agrobiotech Research Centre, Slovak University of Agriculture in Nitra, 94976 Nitra, Slovakia; michal.duracka@uniag.sk; 5 Research and Graduate Studies, Mohammed Bin Rashid University of Medicine and Health Sciences, Dubai Health, Dubai P.O. Box 505055, United Arab Emirates

**Keywords:** sperm cryopreservation, cryoresistance, good freezers, poor freezers, thermoresistance, candidate indicators

## Abstract

**Highlights:**

**What are the main findings?**

- After freezing and thawing, semen from good freezers loses less function than poor freezers across movement, membrane health, mitochondrial energy and DNA quality, holding up better during a thermoresistance test at 37 °C.
- A small panel of pre-freeze assays (JC-1, hypo-osmotic swelling test, *Pisum sativum* lectin, merocyanine) and exploratory proteins associated with the tail and acrosome (proAKAP4, AKAP4, SPAG6, HSP70, proacrosin, acrosin) may serve as candidate indicators associated with post-thaw cryoresistance and support future predictive model development.

**What is the implication of the main finding?**

- In the long term, laboratories may use validated pre-freeze screening strategies to adjust cryopreservation handling, prioritize samples, reduce waste and provide patients with clearer expectations about post-thaw quality.

**Abstract:**

Sperm cryopreservation is essential for fertility preservation and assisted reproduction; however, post-thaw sperm quality varies markedly between individuals despite standardized protocols. This exploratory study investigated whether good and poor freezers differ in cellular, sub-cellular, and molecular features associated with cryoresistance. One ejaculate from each of 100 normozoospermic donors was assessed before cryopreservation and after thawing. Samples were classified after thawing as good freezers (GFs; *n* = 50) or poor freezers (PFs; *n* = 50) according to post-thaw motility performance, using total motility ≥ 42% and progressive motility ≥ 30% as thresholds. Sperm quality was evaluated using computer-assisted sperm analysis, membrane and acrosome integrity assays, mitochondrial membrane potential, capacitation-associated patterns, membrane lipid disorder and DNA/chromatin integrity tests. Post-thaw thermoresistance was assessed at 37 °C for 60 and 120 min. An exploratory Western blot panel was performed only on fresh pre-freeze samples from a selected subset of 4 GF and 4 PF samples. Cryopreservation reduced sperm quality in both groups; however, PF samples showed significantly greater declines in total and progressive motility, sperm viability, membrane and acrosome integrity, mitochondrial membrane potential and thermoresistance. Exploratory Western blot data showed GF-favoring patterns for proAKAP4, SPAG6, proACR and the proACR/ACR ratio, whereas PF samples showed relatively higher ACR abundance; these molecular findings require validation in larger cohorts. Our data indicate that human sperm cryoresistance is a coordinated multi-level phenomenon involving membrane, mitochondrial, acrosomal, chromatin and flagellar resilience. This study identifies candidate functional and molecular indicators associated with cryoresistance and supports future development of pre-freeze screening strategies rather than providing a validated predictive model or clinical cut-off values.

## 1. Introduction

Human sperm cryopreservation underpins fertility preservation, assisted reproduction logistics, and donor banking [1,2], yet post-thaw quality is notoriously variable across individuals despite standardized protocols [3]. Freeze–thaw cycles may compromise membrane architecture, axonemal function, mitochondria and nuclear chromatin, leading to a consistent decline of the sperm motility and viability alongside increased sperm DNA fragmentation, all of which has been consistently documented across cohorts and media [4]. This underlying intra-species variability drives efforts to identify pre-freeze predictors of sperm cryoresistance that may anticipate post-thaw resilience of male gametes and inform clinical decision-making.

Cryopreservation exposes spermatozoa to a sequence of osmotic, thermal and oxidative stresses that may impair several cellular compartments simultaneously. In human spermatozoa, freezing and thawing have been associated with reduced motility, altered plasma membrane fluidity and permeability, acrosomal destabilization, mitochondrial dysfunction, premature capacitation-like changes and increased susceptibility to DNA and chromatin damage. Because these features are biologically interconnected, post-thaw sperm survival is unlikely to depend on a single parameter alone. Individual cryoresistance may therefore reflect the combined pre-freeze status of the plasma membrane, mitochondria, acrosome, chromatin and flagellar structures [1,4].

In practice, clinics often see men who provide excellent ejaculates for immediate insemination or in vitro fertilization (IVF) but whose post-thaw survival collapses, while others remain robust, which are by and large patterns that routine baseline semen analysis does not or cannot fully explain. As such, a recently emerging and overarching goal of the andrology research community is to identify cellular, sub-cellular and molecular characteristics that could pre-determine a given individual’s tolerance to low temperatures and thereby predict the cryo-viability of such sample prior its processing [3]. This concept assumes that cryodamage is not purely a procedural artifact but is modulated by genetic/epigenetic control and molecular state, including the composition and organization of intra- and extracellular molecules that shape the sperm response to cold stress [4].

From a clinical perspective, such a practical predictor should be bench-friendly and mechanistically anchored. On the functional side, mitochondrial activity, osmotic/membrane integrity, acrosome status and merocyanine membrane lipid configuration summarize states known to affect freeze–thaw outcomes and, in prior works, have related to post-thaw function or fertilization potential [5,6,7,8,9]. Complementarily, a brief 37 °C thermoresistance challenge can unmask a latent sperm fragility and thus could serve as a sensitive quality-control stress test following the thawing process, irrespective of a direct fertility prediction [10,11].

To add a molecular perspective, it may be plausible to assume that fluctuations may be observed in proteins with direct roles in motility and fertilization: A-kinase anchoring protein 4 (AKAP4), a principal-piece fibrous-sheath scaffold supporting sustained motility (with proAKAP4 increasingly reported as a quantitative marker of semen fitness across species) [12,13]; sperm-associated antigen 6 (SPAG6), a central-apparatus component critical for flagellar beat [14]; heat shock protein 70 (HSP70), a stress chaperone implicated in protecting membranes and bioenergetics during cooling and freezing [15]; as well as acrosin (ACR) and its acrosomal zymogen proacrosin (proACR), both essential for zona pellucida penetration [16]. Given their biological roles, these markers were selected as candidate proteins potentially associated with cryoresistance.

In this study, we integrated (a) pre-freeze/post-thaw functional sperm characterization; (b) a 37 °C thermoresistance test (60/120 min); and (c) a targeted Western blot panel (proAKAP4, AKAP4, SPAG6, HSP70, proACR, ACR) to test two hypotheses:

1. Semen samples classified after thawing as good vs. poor freezers may diverge on within-sample change (Delta = Frozen − Fresh) and Recovery% across motility, membrane/acrosome integrity, mitochondrial function, capacitation patterns, DNA status and thermoresistance.
2. A compact pre-freeze screening set combining functional outputs with selected target proteins may identify candidate indicators associated with the extent of post-thaw decline in an individual sample and thus support future predictive model development and informed semen handling before cryopreservation.

## 2. Materials and Methods

### 2.1. Study Design and Sample Collection

This was a prospective laboratory study conducted at the Mohammed Bin Rashid University of Medicine and Health Sciences (MBRU), Dubai (UAE). All procedures adhered to institutional and national guidelines and the Declaration of Helsinki. Ethics approval was obtained from the Institutional Ethics Committee of the Mohammed Bin Rashid University of Medicine and Health Sciences (Approval No. MBRU IRB-2021-12). All donors provided a written informed consent.

Ejaculates were obtained from normozoospermic volunteers (25–43 years old) attending the andrology laboratory at the Dubai Fertility Centre in Dubai (UAE) and processed according to WHO criteria [17]. Inclusion criteria: ejaculatory abstinence of 2–3 days; no febrile illness in the past month; no known reproductive tract infection or varicocele; no gonadotoxic medication. The samples were allowed to liquefy at 37 °C for 20–30 min before baseline analyses.

All ejaculates were split into two aliquots: fresh samples and cryopreserved samples for subsequent paired comparison. Each donor contributed one ejaculate only; hence, the final dataset consisted of 100 independent specimens from 100 individual donors. All quality assessments (except Western blotting) were carried out in fresh specimens as well as their frozen–thawed counterparts. Samples were allocated to GF or PF groups only after thawing, according to post-thaw motility performance under the applied cryopreservation protocol. The classification was anchored to the World Health Organization sixth edition lower reference values for human semen motility, namely, total motility ≥ 42% and progressive motility ≥ 30%. Samples retaining post-thaw total motility ≥ 42% and progressive motility ≥ 30% were classified as good freezers (GFs; *n* = 50), whereas samples falling below these thresholds were classified as poor freezers (PFs; *n* = 50).

### 2.2. Reagents and Media

All reagents were purchased from Sigma Aldrich (St. Louis, MO, USA) unless otherwise specified. All routine media were sterile and equilibrated to experimental temperature before use.

### 2.3. Sperm Cryopreservation

For the freezing procedure, spermatozoa were washed (300× *g*, 5 min) and resuspended in the G-Gamete medium (Vitrolife, Gothenburg, Sweden). SpermFreeze™ (FertiPro, Beernem, Belgium) was slowly added to reach 70% (*v*/*v*) of the final volume. The samples were then equilibrated at 4 °C for 15–20 min, exposed to liquid nitrogen (LN_2_) vapor (~−80 °C) for 15–20 min, and plunged into LN_2_. Frozen specimens were kept in LN_2_ for at least for one month [18,19].

Thawing was performed in a water bath at 37 °C for 2 min, followed by the removal of the cryopreservation medium. Spermatozoa were washed twice (300× *g*, 5 min) in sperm-washing medium (Fujifilm Irvine Scientific, Santa Ana, CA, USA) and resuspended in protein-free HAM-HEPES-F10 medium (Gibco, Thermo Fisher, Waltham, MA, USA) [18]. After thawing and washing, all samples were equilibrated for 30 min at 37 °C prior to subsequent assays.

### 2.4. Sperm Motility Parameters

Total (TMOT) and progressive motility (PMOT), straight-line velocity (VSL), amplitude of lateral head displacement (ALH), linearity (LIN), and beat cross frequency (BCF) were measured using a computer-assisted sperm analysis (CASA) system (Sperm Class Analyzer version 5.4-SCA^®^; Microptic, S.L., Barcelona, Spain,) equipped with a Basler A312fc digital color camera (Microptic, S.L., Barcelona, Spain). Leja slides (20 µm chamber depth; Leja Products B.V., Nieuw-Vennep, The Netherlands) were pre-warmed to 37 °C and used for sample loading. The system was set up at ×200 magnification, 25 frames/s, 25 frames/track, chamber depth 20 µm, and 37 °C heated stage. Progressive motility was defined as VAP ≥ 25 µm/s and STR ≥ 80% [19].

### 2.5. Thermoresistance Test

Post-thaw thermoresistance was assessed to evaluate the functional endurance of spermatozoa following cryopreservation. After thawing and washing, sperm samples were incubated at 37 °C, and motility was reassessed after 60 and 120 min of incubation. Total motility (TMOT) and progressive motility (PMOT) were evaluated using CASA under the same settings as used for the standard motility assessment [19,20].

### 2.6. Sperm Membrane Stability, Viability and Acrosome Integrity

Sperm plasma membrane integrity was further assessed using a fluorescent staining protocol based on carboxyfluorescein diacetate (CFDA) and DAPI. All samples were normalized to 1 × 10^6^ spermatozoa prior to staining. A 100 µL sperm suspension was mixed with 10 µL CFDA solution prepared at 0.75 mg/mL in DMSO, resulting in a final CFDA concentration of 68.2 µg/mL. A total of 10 µL DAPI (1 µM in PBS; final concentration 0.091 µM) was added for nuclear counterstaining. Samples were incubated for 15 min at 37 °C in the dark, centrifuged, washed twice with PBS, and evaluated by fluorescence assessment. Results are expressed as the percentage of CFDA-live spermatozoa [21,22].

Functional plasma membrane integrity was assessed using the hypo-osmotic swelling test (HOST). The hypo-osmotic solution was prepared by dissolving 0.735 g sodium citrate dihydrate and 1.351 g D-fructose in 100 mL distilled water. The osmolarity of the solution was verified using an osmometer and confirmed at 150 mOsm/kg. Sperm aliquots were incubated in the HOST solution at 37 °C and evaluated no earlier than 30 min and no later than 60 min after exposure. Spermatozoa showing characteristic tail swelling or coiling were classified as HOST-positive. At least 200 spermatozoa were evaluated per sample under a light microscope at 100× magnification, and the results are expressed as the percentage of HOST-positive spermatozoa [23].

Acrosome integrity was evaluated using fluorescein isothiocyanate-conjugated peanut agglutinin (FITC-PNA). Briefly, 100 µL sperm suspension containing 1 × 10^6^ spermatozoa was mixed with 100 µL FITC-PNA working solution prepared at 10 µM in PBS, resulting in a final FITC-PNA concentration of 5 µM, and DAPI was used as a nuclear counterstain as described above. Samples were incubated for 30 min at 37 °C in the dark. At least 300 spermatozoa per sample were examined using an epifluorescence microscope at 40× magnification. Spermatozoa negative for PNA staining were classified as acrosome-intact, and results are expressed as the percentage of PNA-intact spermatozoa [21].

Sperm phosphatidylserine externalization and plasma membrane integrity were evaluated using the commercially available Annexin-V-FLUOS kit (Roche Applied Science, Basel, Switzerland) according to the manufacturer’s instructions. Propidium iodide (PI), supplied as part of the kit, was used to distinguish membrane-intact from membrane-disrupted spermatozoa. Briefly, sperm suspensions were adjusted with pre-warmed phosphate-buffered saline (PBS) to 1 × 10^6^ cells. Each aliquot was incubated with 50 µL incubation buffer, 1 µL Annexin V-FLUOS reagent and 1 µL PI for 30 min at 37 °C in the dark. DAPI was used as a nuclear counterstain as described above. At least 300 spermatozoa per sample were evaluated using an epifluorescence microscope equipped with a 40× objective (Leica Microsystems, Wetzlar, Germany). Spermatozoa were classified as AV^−^/PI^−^ viable/intact cells; AV^+^/PI^−^ early apoptotic-like cells with phosphatidylserine externalization and preserved plasma membrane integrity; AV^+^/PI^+^ late apoptotic-like or membrane-disrupted cells; and AV^−^/PI^+^ necrotic/membrane-disrupted cells. The Annexin V endpoint reported in this study corresponds to AV^+^/PI^−^ cells (%), while PI-positive spermatozoa (%) were reported separately.

Membrane lipid disorder was assessed using merocyanine 540 (M540). Sperm suspensions were mixed 1:1 with M540 working solution, resulting in a final M540 concentration of 2.7 µM, and incubated for 15 min at 37 °C in the dark. After incubation, spermatozoa were evaluated under an epifluorescence microscope at 40×, and cells showing high M540 fluorescence were classified as merocyanine-high. At least 200 spermatozoa were evaluated per sample, and results are expressed as the percentage of M540-high spermatozoa [5].

### 2.7. Sperm Mitochondrial Activity, Capacitation Status, and Chromatin Integrity

Mitochondrial membrane potential was examined using the JC-1 Mitochondrial Membrane Potential Assay Kit (Cayman Chemical, Ann Arbor, MI, USA). Sperm aliquots containing 1 × 10^6^ cells were adjusted to 100 µL with PBS and stained with 5 µL of 5 µM JC-1 working solution, resulting in a final JC-1 concentration of 0.238 µM. Following incubation at 37 °C for 20 min in the dark, cells were centrifuged at 150× *g* for 10 min at 20 °C and washed twice with PBS. The final suspension was transferred to a black 96-well plate, and JC-1 monomeric and aggregate fluorescence signals were quantified using a Glomax Multi+ combined spectro-fluoro-luminometer. Results are expressed as the percentage of spermatozoa with high mitochondrial membrane potential [21].

Capacitation-associated membrane changes were evaluated using the chlortetracycline fluorescence assay (CTC). Briefly, 45 µL sperm suspension was mixed with 45 µL freshly prepared 750 µM CTC solution, resulting in a final CTC concentration of 375 µM. Spermatozoa were evaluated using an epifluorescence microscope at 40×. Based on fluorescence distribution, three patterns were identified: F pattern, characterized by uniform fluorescence over the sperm head and interpreted as non-capacitated spermatozoa; B pattern, characterized by fluorescence in the post-acrosomal region and interpreted as capacitated spermatozoa; and AR pattern, characterized by loss of acrosomal fluorescence and interpreted as acrosome-reacted spermatozoa. At least 200 spermatozoa were evaluated per sample, and results are expressed as the percentage of spermatozoa showing each CTC pattern [24].

Sperm chromatin dispersion (SCD) was assessed using the Halosperm^®^ commercial kit (Halotech DNA, Madrid, Spain). Briefly, 20 µL semen suspension was mixed with low-melting-point agarose, placed onto agarose-precoated microscope slides, and cooled at 4 °C until solidified. Slides were then exposed to acid denaturation solution for 7 min and lysis solution for 20 min, washed with distilled water, dehydrated in 70% and 100% ethanol for 2 min each, and air-dried. After staining with SYBR Green (0.7 µg/mL), at least 300 spermatozoa were evaluated under an epifluorescence microscope at 40× magnification. Spermatozoa without a halo or with a small halo were classified as having fragmented DNA, and results are expressed as sperm chromatin dispersion DNA fragmentation index (SCD-DFI, %) [21].

Sperm DNA fragmentation was further assessed using the Apo-DIRECT^TM^ TUNEL kit (Thermo Fisher Scientific, Waltham, MA, USA). Samples were adjusted to 2.5 × 10^6^ spermatozoa, centrifuged at 300× *g* for 10 min at 20 °C, and washed twice with PBS. Spermatozoa were fixed in 4% paraformaldehyde for 15 min, centrifuged, permeabilized in 70% ice-cold ethanol, and stored at −20 °C until analysis. For the assay, cells were washed twice with the wash buffer, incubated with staining solution containing reaction buffer, terminal deoxynucleotidyl transferase enzyme, FITC-dUTP, and distilled water for 60 min at 37 °C, and subsequently rinsed. Finally, PI/RNase solution was added, and samples were transferred to a black 96-well plate for fluorescence assessment using the Glomax Multi+ system. Results are expressed as the percentage of TUNEL-positive spermatozoa [21].

DNA susceptibility to denaturation was assessed using Acridine Orange (AO) staining. Each sample was adjusted to 2 × 10^6^ spermatozoa/mL with TNE buffer (0.15 M NaCl, 0.01 M Tris-HCl, 1 mM EDTA, pH 7.4), mixed with 0.4 mL acid detergent solution (0.17% Triton X-100, 0.15 M NaCl, and 0.08 N HCl, pH 1.4), and incubated for 30 s. The cells were then stained with AO staining solution containing 0.1 M citric acid, 0.2 M Na_2_HPO_4_, 1 mM EDTA, 0.15 M NaCl, and 6 µg/mL AO (pH 6.0). At least 200 spermatozoa were evaluated per sample. Spermatozoa showing green fluorescence were considered to have intact double-stranded DNA, whereas spermatozoa showing yellow-orange to red fluorescence were considered to have denatured or damaged DNA. Results are expressed as the percentage of AO-red spermatozoa [25].

Chromatin maturity was evaluated using Aniline Blue (AB) staining. Fresh sperm smears were air-dried and fixed in 3% buffered glutaraldehyde in 0.2 M phosphate buffer (pH 7.2) for 30 min at room temperature. Slides were then stained with 5% aqueous AB in 4% acetic acid (pH 3.5) for 5 min. At least 200 spermatozoa were assessed per slide using light microscopy. Unstained or pale-blue spermatozoa were considered to have normal chromatin maturity, whereas dark-blue spermatozoa were classified as abnormal, indicating retained lysine-rich histones and incomplete histone-to-protamine replacement. Results are expressed as the percentage of AB-positive spermatozoa [26].

### 2.8. Western Blots

For the exploratory Western blot analysis, only fresh pre-freeze sperm samples were used. The analysis was performed on a selected subset of eight samples, comprising four GF and four PF representatives. The samples were selected based on sufficient sperm concentration for lysis and adequate total protein yield after extraction to ensure reliable protein loading and signal detection. Spermatozoa were centrifuged at 300× *g* for 7 min, and the collected pellets were subjected to RIPA lysis buffer (Merck, Darmstadt, Germany) containing a protease inhibitor cocktail overnight at 4 °C to allow complete cell lysis. After centrifugation at 13,000× *g* for 30 min, the supernatant was aspirated and subjected to protein concentration determination using the Pierce^TM^ BCA protein assay kit (Thermo Fisher Scientific, Waltham, MA, USA). Reagent blanks and RIPA buffer blanks were included for background correction, and absorbance values were blank-corrected before calculating protein concentration from the standard curve. All samples were adjusted using PBS to reach a uniform concentration of 25 µg protein. The samples were then treated with 4× Laemmli buffer (Bio-Rad, Hercules, CA, USA) and beta-mercaptoethanol and subsequently boiled at 95 °C for 10 min. The samples were loaded (20 µL) into Mini-PROTEAN TGX stain-free polyacrylamide gels (Bio-Rad, Hercules, CA, USA), together with 7 µL Precision Plus Protein marker (Bio-Rad, Hercules, CA, USA). SDS-polyacrylamide gel electrophoresis was run at 110 V for 1 h, and the gels were visualized with the ChemiDoc Imaging System (Bio-Rad, Hercules, CA, USA) to confirm loading uniformity. The gels were then transferred to polyvinylidene difluoride membranes (Trans-Blot Turbo Pack; Bio-Rad, Hercules, CA, USA) using the Trans-Blot^®^ Semi-Dry Electrophoretic Transfer Cell (Bio-Rad, Hercules, CA, USA) at 18 V and 2.5 A for 30 min. The resulting membranes were incubated for 3 × 10 min in Tris-buffered saline (TBS) and blocked with 5% skim milk (Blotting grade blocker; Bio-Rad, Hercules, CA, USA) in TBS containing 0.1% Tween-20 for 1 h. Finally, the membranes were incubated with selected primary antibodies (listed in Table 1) diluted 1:1000 in 5% skim milk in TBS/0.1% Tween-20 at 4 °C overnight [27].

The next day, the membranes were washed for 5 × 10 min in 1% milk in TBS/0.2% Tween-20 and subsequently incubated with a secondary anti-mouse antibody (for proAKAP4 and SPAG6) (GE Healthcare, Chicago, IL, USA) or anti-rabbit antibody (for HSP70 and ACR) (GE Healthcare, Chicago, IL, USA) diluted 1:15,000 in 1% milk in TBS/0.2% Tween-20 for 1 h. Following incubation, the membranes were washed for 3 × 10 min in TBS/0.2% Tween-20 at room temperature. To visualize the target proteins, membranes were incubated with ECL substrate (GE Healthcare, Chicago, IL, USA) in the dark for 5 min. Proteins were visualized with the ChemiDoc Imaging System and quantified using Image Lab software (version 6.1; Bio-Rad, Hercules, CA, USA). Only non-saturated images were used for quantification, and target band intensities were normalized to TGX stain-free total lane protein [27].

The anti-proAKAP4 antibody detected two AKAP4-immunoreactive bands corresponding to the precursor form, proAKAP4, and the mature AKAP4 protein. Similarly, the anti-acrosin antibody detected two ACR-immunoreactive bands corresponding to proACR and processed ACR. These bands were quantified separately according to their expected molecular weights. HSP70 and SPAG6 were quantified as individual immunoreactive bands.

### 2.9. Statistical Analysis

The collected data were processed with GraphPad Prism for Mac (version 11.0.2; GraphPad Software; San Diego, CA, USA) and are presented as mean ± standard deviation (SD). Descriptive statistics were calculated for each sperm quality parameter separately in GFs and PFs before cryopreservation and after thawing. Cryo-induced changes were quantified using within-sample delta values, calculated as Frozen − Fresh, and Recovery%, calculated as 100 × Frozen/Fresh. Differences between GFs and PFs in delta values and Recovery% were assessed using Welch’s unpaired *t*-test. Effect sizes were reported as Hedges g. To account for multiple testing, Benjamini–Hochberg false discovery rate correction was applied, and adjusted *p*-values are reported as q-values. Nominal *p*-values and FDR-adjusted q-values are reported; q < 0.05 was considered significant after multiple-testing correction. Values below 0.0001 were reported as <0.0001.

For the thermoresistance assay, post-thaw total and progressive motility were evaluated after incubation at 37 °C for 60 and 120 min in all 50 GF and 50 PF samples. Area under the curve from 60 to 120 min was calculated using the trapezoidal rule and compared between GFs and PFs using Welch’s unpaired *t*-test.

Western blot-derived protein abundance was analyzed in a technically selected exploratory subset of fresh pre-freeze samples from four GF and four PF samples. Protein signals were normalized to TGX stain-free total lane protein, and GF-PF comparisons were performed using Welch’s unpaired *t*-test with Hedges g effect size estimation. Because of the small sample size, the Western blot results were interpreted as exploratory and hypothesis-generating.

## 3. Results

### 3.1. Fresh and Frozen–Thawed Sperm Quality in Good and Poor Freezers

The descriptive sperm quality characteristics of GFs and PFs before and after cryopreservation are provided in Supplementary Table S1. In the fresh state, GFs and PFs differed moderately across several parameters, with PF samples generally showing lower functional quality already before freezing. However, differences between the two groups became more pronounced following the cryopreservation procedure.

The CASA analysis showed that both groups exhibited a decline in motility after freezing and thawing, but the loss was substantially greater in PF samples. TMOT decreased from 72.98 ± 5.72% to 48.00 ± 7.66% in GFs, whereas PFs decreased from 69.04 ± 5.65% to 26.30 ± 7.83%. PMOT followed the same pattern, declining from 58.56 ± 5.87% to 35.56 ± 3.75% in GFs and from 54.02 ± 6.20% to 15.86 ± 4.76% in PFs. Similar GF-favorable post-thaw kinetics patterns were observed for VAP, VSL, LIN, ALH, and BCF.

Membrane and acrosomal integrity markers also showed stronger post-thaw preservation in GFs. After cryopreservation, GFs retained higher CFDA-live spermatozoa, HOST-positive spermatozoa, and PNA-intact acrosomes than PFs. Conversely, PF samples presented with higher post-thaw AV-positive and PI-positive spermatozoa, indicating greater membrane destabilization and loss of viability. Merocyanine-high spermatozoa were also more abundant in PFs after freezing, suggesting an increased membrane lipid disorder.

Mitochondrial membrane potential was better preserved in GFs, as shown by a higher post-thaw proportion of JC1-high spermatozoa. Capacitation-associated CTC patterns showed that PF samples had a stronger post-thaw shift toward the CTC-AR pattern. DNA and chromatin markers also showed an unfavorable PF profile, with higher frozen–thawed values for SCD, TUNEL-positive, AO-red and AB-positive spermatozoa. Overall, the descriptive data indicate that PF specimens underwent a broader and more severe cryo-induced deterioration than GF samples.

### 3.2. Cryo-Induced Change and Recovery Differences Between GF and PF Semen Samples

To quantify the magnitude of cryo-induced change, within-sample delta values were calculated as Frozen − Fresh, while Recovery% was calculated as 100 × Frozen/Fresh. These results are presented in Table 2. Across most endpoints, GFs showed smaller cryo-induced deterioration and higher recovery than PFs.

For the CASA outputs, GFs had significantly smaller declines in total and progressive motility. TMOT decreased by −25.02 ± 10.74 percentage points in GFs compared with −42.74 ± 11.74 percentage points in PFs, corresponding to significantly higher recovery in GFs than PFs. The difference was even more pronounced for PMOT, where GFs retained 61.42 ± 9.44% of the fresh value, while PFs retained only 30.17 ± 11.50%. VAP and VSL also showed significantly better preservation in GFs, while LIN and ALH showed smaller but still significant GF-favorable differences. BCF did not clearly discriminate the two freezer groups, and its Recovery% showed high variability.

In contrast, PF samples showed stronger increases in PI-positive and AV^+^/PI^−^ spermatozoa, consistent with greater cryo-induced membrane damage and phosphatidylserine externalization. For AV^+^/PI^−^ and PI-positive spermatozoa, delta values were more informative than Recovery%, because these endpoints represent damage-associated increases rather than beneficial retained function.

Acrosomal integrity was also strongly associated with freezer phenotype. PNA-intact spermatozoa declined less in GFs than PFs, and PNA recovery was markedly higher in GFs. In parallel, PFs showed a greater increase in CTC-AR spermatozoa, while CTC-F and CTC-B were less discriminatory. The pronounced increase in CTC-AR in PFs suggests stronger cryo-induced capacitation-like or acrosome-reacted shifts in cryosensitive samples.

Merocyanine-high spermatozoa increased in both groups, but the increase was substantially greater in PFs. This indicates that PF samples underwent stronger membrane lipid disorder following cryopreservation. Mitochondrial membrane potential showed a GF-favorable pattern, with JC1-high recovery substantially higher in GFs than PFs, even though both groups experienced a post-thaw decline.

DNA and chromatin integrity endpoints also supported a stronger cryo-induced injury pattern in PFs. TUNEL-positive spermatozoa increased more in PFs than GFs, and SCD also showed a larger delta increase in PFs. AO-red and Aniline Blue-positive spermatozoa similarly increased after freezing, with generally greater deterioration in PFs. These findings suggest that poor freezer status is associated not only with impaired motility and membrane function but also with greater chromatin and DNA vulnerability.

Taken together, the delta and recovery analyses show that GFs and PFs differ across multiple biological domains. The strongest and most consistent GF-favorable effects were observed for progressive motility recovery, CFDA-live recovery, HOST-positive recovery, PNA-intact recovery, CTC-AR delta, merocyanine-high delta, and JC1-high recovery.

### 3.3. Post-Thaw Thermoresistance Indicates Greater Functional Endurance in GF Spermatozoa

Post-thaw thermoresistance at 37 °C is presented in Table 3. GF samples maintained a substantially higher total and progressive motility than PF samples at both 60 and 120 min.

The AUC analysis confirmed the pronounced difference in post-thaw motility endurance. TMOT AUC was substantially higher in GFs than PFs, with a very large effect size. Similarly, PMOT AUC was markedly higher in GFs than PFs. These results indicate that GF spermatozoa were not only more resistant to the immediate effects of freezing and thawing but also maintained functional stability more effectively during post-thaw incubation at physiological temperature.

### 3.4. Exploratory Western Blot Analysis of Candidate Cryoresistance-Associated Proteins

**Figure 1:**
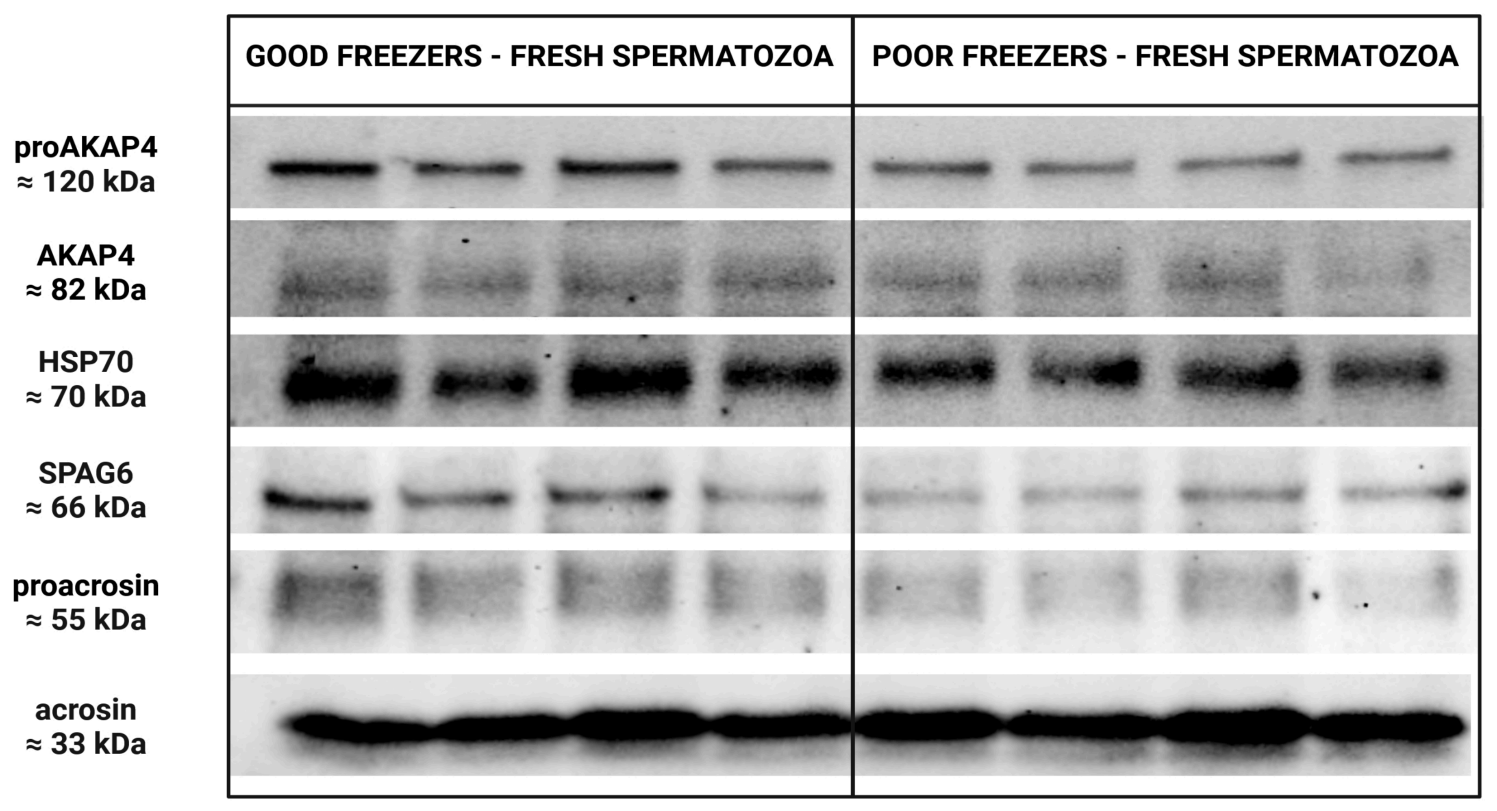
Representative Western blot profile of candidate cryoresistance-associated proteins in fresh spermatozoa from good and poor freezers.

Western blot results are summarized in Table 4, and representative Western blot profiles are shown in Figure 1. Although four primary antibodies were used for multiplex immunoblotting, six immunoreactive protein bands were quantified. The anti-AKAP4 antibody enabled separate quantification of proAKAP4 and mature AKAP4, while the anti-acrosin antibody enabled separate quantification of proACR and ACR. HSP70 and SPAG6 were quantified as single bands. All bands were normalized to TGX stain-free total lane protein.

Across the flagellar markers, GF samples showed higher mean abundance of proAKAP4 and SPAG6 than PF samples, both with large effect sizes. AKAP4 and HSP70 showed smaller GF-favoring differences. Within the acrosomal panel, GF samples showed higher proACR abundance, whereas PF samples showed higher ACR abundance. Consequently, the proACR/ACR ratio was markedly higher in GFs than PFs. Although several comparisons reached nominal *p* < 0.05, none remained significant after FDR correction across the exploratory WB panel. These data nevertheless suggest that GF spermatozoa may enter cryopreservation with a more favorable flagellar and acrosomal protein profile, while PF samples may show a shift toward increased proACR processing.

Representative cropped immunoblot bands showed proAKAP4, AKAP4, HSP70, SPAG6, proACR and ACR in fresh pre-freeze spermatozoa from good freezers and poor freezers. Four biological replicates are shown per group. ProAKAP4 and AKAP4 were detected as separate AKAP4-immunoreactive bands using the same anti-proAKAP4 antibody, while proACR and ACR were detected as separate acrosin-immunoreactive bands using the same anti-ACR antibody. HSP70 and SPAG6 were detected as individual immunoreactive bands. Band intensities were quantified from non-saturated ChemiDoc images using Bio-Rad Image Lab software (version 6.1) and normalized to TGX stain-free total lane protein. Original photographs of the gels and blots are available in the Supplementary Materials. Created with BioRender.com (accessed on 22 June 2026).

## 4. Discussion

The present study demonstrates that human sperm cryoresistance is reflected by a coordinated, multi-level phenotype involving motility, membrane and acrosomal stability, mitochondrial function, capacitation-associated changes, DNA/chromatin integrity, and post-thaw thermoresistance. Although all samples were processed using the same cryopreservation protocol, good freezers (GFs) retained substantially more functionality after thawing than poor freezers (PFs). This supports the concept that post-thaw survival is not determined exclusively by the freezing protocol but also by intrinsic cellular, sub-cellular and molecular properties of spermatozoa that differ between individuals [3].

The clearest separation between GFs and PFs was observed in CASA-derived motility. GF samples showed a higher degree of recovery of total and progressive motility, whereas PF samples exhibited substantially greater post-thaw losses. This agrees with previous studies emphasizing that cryopreservation impairs sperm motility, viability, morphology, and mitochondrial function [18,19,27,28,29,30]. However, our data also suggest that motility loss is only one aspect of a broader cryosensitivity manifestation. The parallel deterioration of membrane and acrosome integrity, mitochondrial function and chromatin stability indicates that poor post-thaw motility likely reflects a deeper structural and biochemical vulnerability rather than a single flagellar alteration.

Membrane-related markers were among the strongest discriminators of freezer phenotype, agreeing with a previously postulated paradigm on the central role of the sperm plasma membrane in cryoinjury. Cooling, freezing, and thawing have been repeatedly shown to disturb the lipid organization, membrane permeability, osmotic balance and membrane-associated signaling [31], which may explain why markers of membrane integrity and lipid disorder separated GFs and PFs so clearly in this study. The merocyanine pattern is particularly relevant since an increased M540 signal reflects membrane lipid disorder [5] and may indicate cryo-induced membrane destabilization. Together with the CTC results, this suggests that PF spermatozoa are more prone to cryo-induced membrane remodeling and premature capacitation-like or acrosome-reacted shifts [5,24,32,33]. Nevertheless, Annexin V and merocyanine positivity in human spermatozoa should be interpreted cautiously, as these markers may reflect early membrane degeneration and altered phospholipid asymmetry rather than classical apoptosis or capacitation alone [5].

The HOST results should be interpreted in the same functional context. HOST does not measure the same biological property as CASA-derived total motility and should not be considered numerically interchangeable with total viable or motile spermatozoa. In this study, HOST-positive spermatozoa represented the fraction able to respond to hypo-osmotic stress with characteristic tail swelling or coiling, reflecting osmotic membrane responsiveness rather than viability alone. Therefore, HOST values may be lower than TMOT, particularly after cryopreservation, because a motile spermatozoon may still show impaired osmotic membrane responsiveness after freeze–thaw-induced membrane stress.

Mitochondrial function also emerged as an important component of cryoresistance. GF samples preserved a higher proportion of JC-1-high spermatozoa, whereas PF samples showed stronger mitochondrial depolarization following thawing. This is consistent with previous reports showing that cryopreservation reduces the sperm mitochondrial activity, which is then closely associated with the loss of motility and viability [30,34,35]. Since mitochondria contribute to energy regulation, redox balance, and flagellar activity, their preservation may be essential for maintaining post-thaw function. In this context, the 37 °C thermoresistance test was particularly informative. GF samples not only survived thawing more effectively but also maintained total and progressive motility during incubation at physiological temperature. As such, thermoresistance appears to capture post-cryopreservation functional endurance that may not be fully reflected by a single immediate post-thaw CASA assessment [11,20].

DNA and chromatin parameters further support the presence of an intrinsic cryosensitivity profile, as PF samples showed larger increases in DNA damage across all assessed markers. These findings are in line with previous studies reporting that conventional sperm freezing may affect the integrity of the paternal genome [10,28,36]. Importantly, these assays do not measure identical biological phenomena. Whilst TUNEL and SCD reflect DNA fragmentation or chromatin dispersion, Aniline Blue detects retained lysine-rich histones and incomplete histone-to-protamine replacement, and Acridine Orange reflects susceptibility of DNA to denaturation. As such, we may speculate that the post-thaw increase in Aniline Blue positivity does not reflect newly induced histone retention or active chromatin decondensation during storage. Rather, freeze–thaw stress may increase the accessibility of histone-rich or insufficiently protaminated chromatin regions, thereby revealing pre-existing chromatin packaging vulnerability. This interpretation is consistent with the parallel increases in SCD, TUNEL positivity, and AO-red fluorescence, suggesting that cryopreservation exposes or amplifies underlying chromatin instability in cryosensitive samples [37,38].

Mechanistically, the convergence of membrane, mitochondrial, capacitation-associated and DNA attributes suggests that the PF phenotype may reflect a reduced capacity to buffer the combined osmotic, thermal and oxidative stresses triggered by freezing and thawing [4]. Temperature reduction and ice crystal formation disrupt the spatial organization of membrane proteins and ion channels, which may increase membrane permeability, disturb calcium homeostasis and promote capacitation-like membrane remodeling [39]. Mitochondrial depolarization may further exacerbate this process by reducing the energetic support for flagellar movement and contributing to redox imbalance. A parallel rise in the sperm DNA damage in PF samples furthermore suggests that chromatin packaging and DNA stability may determine how vulnerable sperm nuclei are to this stress cascade [18,38]. As such, the GF phenotype may be interpreted as a state of higher structural and bioenergetic resilience even before freezing, whereas the PF phenotype may reflect a pre-existing susceptibility that may become unmasked during cryopreservation.

The exploratory Western blot data provide preliminary molecular support for this interpretation. Although the analysis was limited to fresh pre-freeze samples from a limited subset of four GF and four PF samples, several biologically coherent patterns emerged. GF samples showed higher mean abundance of proAKAP4 and SPAG6, both with large effect sizes, suggesting more favorable flagellar structural readiness. AKAP4 and HSP70 showed smaller GF-favoring differences. The acrosomal profile was particularly informative: GF samples showed higher proACR abundance, whereas PF samples showed relatively higher ACR abundance, resulting in a higher proACR/ACR ratio in GF. Because proACR is the zymogen precursor of acrosin, this pattern may indicate preservation of the acrosomal proenzyme reserve in GF and increased proACR processing or premature acrosomal destabilization in PFs [16,38,39]. This interpretation is consistent with the functional data, where PF samples showed lower PNA-intact acrosomes and a larger post-thaw increase in CTC-AR patterns. Since none of the Western blot comparisons remained significant after FDR correction, these signals should be viewed as candidate molecular markers requiring validation. Thus, the Western blot findings support a hypothesis that cryoresistance may be partly associated with the pre-freeze structural readiness of the flagellum-mitochondria-acrosome axis, but they do not establish an independently validated predictive protein panel.

From a translational perspective, the present results support the future development of a two-tier pre-freeze screening strategy. The first tier could consist of rapid, bench-friendly functional assays such as JC-1, HOST, PNA/PI, CFDA or M540. These assays could identify samples with low mitochondrial potential, poor membrane responsiveness, high membrane lipid disorder or elevated chromatin vulnerability before freezing. The second tier, applied in selected clinical cases, could include targeted protein markers such as AKAP4/proAKAP4 or SPAG6. Such a strategy could help laboratories identify samples at higher risk of poor post-thaw survival and adjust semen handling accordingly, for example, by modifying aliquot allocation, post-thaw use strategy or counselling expectations. However, the present study does not establish clinically applicable cut-off values or a validated prediction model, hence requiring prospective validation before clinical implementation.

Several limitations must be acknowledged. First, the GF/PF classification was assigned after thawing using post-thaw motility performance under the applied cryopreservation protocol, and future studies should harmonize freezer phenotype definitions across laboratories. Second, although the full functional dataset included 100 independent samples, the Western blot analysis was exploratory and based on a small fresh-only cohort; therefore, the protein results should be interpreted as preliminary molecular support rather than definitive evidence of prediction. Third, the study was not designed to establish clinically applicable predictive cut-off values. The proposed markers should therefore be interpreted as candidate pre-freeze indicators associated with cryoresistance. Finally, the study focused on post-thaw sperm quality and thermoresistance but did not include fertilization, embryo development or pregnancy outcomes. The findings therefore support future prediction of cryo-viability and post-thaw functional stability but not clinical ART success. Larger prospective studies are required to validate these candidate markers, define robust threshold values, and determine whether compact pre-freeze screening panels can reliably predict post-thaw sperm survival.

## 5. Conclusions

In conclusion, human sperm cryoresistance appears to be a multi-level phenotype involving coordinated differences in motility persistence, plasma membrane functionality, mitochondrial membrane potential, acrosomal stability, chromatin integrity, and thermoresistance. In the full-sample cohort, GF specimens retained substantially better post-thaw function than PF samples across several complementary parameters. Exploratory Western blot analysis further suggested that selected flagellar and acrosomal proteins, particularly proAKAP4, SPAG6, proACR and the proACR/ACR balance, may represent candidate molecular indicators associated with cryoresistance. However, because the protein analysis was performed in a small, selected subset, these findings should be interpreted as hypothesis-generating. Larger prospective studies are required to validate these candidate markers, define robust cut-off values and determine whether compact pre-freeze screening panels can reliably predict post-thaw sperm survival.

## Figures and Tables

**Table 1:** Primary antibodies used for the Western blot analysis.

Antibody	ID	Source	Clonality	Manufacturer
Anti-proAKAP4 antibody (clone 6F12)	4BDX-1701S	mouse	monoclonal	4BioDx (Lille, France)
SPAG6 Antibody (XX-2)	sc-100886	mouse	monoclonal	Santa Cruz Biotechnology (Dallas, TX, USA)
Anti-Hsp70 antibody [EPR16892]	ab181606	rabbit	monoclonal	Abcam (Cambridge, UK)
Acrosin Rabbit Polyclonal Antibody	orb1478	rabbit	polyclonal	Biorbyt Ltd. (Cambridge, UK)

**Table 2:** Cryo-induced change and recovery of sperm quality endpoints in good and poor freezers.

Endpoint	Metric	GF	PF	GF-PF	Welch *t*	Hedges g	*p*-Value	*q*-Value
TMOT	Delta	−25.02 ± 10.74	−42.74 ± 11.74	17.76	7.879	1.564	<0.0001	<0.0001
	Recovery%	66.39 ± 12.37	38.88 ± 14.44	27.51	10.221	2.028	<0.0001	<0.0001
PMOT	Delta	−22.97 ± 7.11	−38.16 ± 8.95	15.16	9.378	1.861	<0.0001	<0.0001
	Recovery%	61.42 ± 9.44	30.17 ± 11.5	31.23	14.856	2.948	<0.0001	<0.0001
VAP	Delta	−16.67 ± 8.25	−29.40 ± 12.00	12.73	6.191	1.228	<0.0001	<0.0001
	Recovery%	77.26 ± 10.79	58.55 ± 16.20	18.71	6.794	1.348	<0.0001	<0.0001
VSL	Delta	−13.06 ± 7.25	−22.26 ± 6.13	9.21	6.847	1.359	<0.0001	<0.0001
	Recovery%	75.44 ± 12.94	55.65 ± 10.35	19.78	8.440	1.675	<0.0001	<0.0001
LIN	Delta	−5.16 ± 4.95	−9.92 ± 8.45	4.76	3.430	0.681	0.0009	0.0015
	Recovery%	91.73 ± 8.02	83.78 ± 13.96	7.94	3.488	0.692	0.0008	0.0011
ALH	Delta	−0.58 ± 0.77	−1.02 ± 0.63	0.44	3.078	0.611	0.0027	0.0036
	Recovery%	86.24 ± 17.99	73.59 ± 15.15	12.64	3.799	0.754	0.0002	0.0004
BCF	Delta	−3.95 ± 4.87	−4.19 ± 3.31	0.24	0.289	0.057	0.7732	0.7732
	Recovery%	102.67 ± 130.12	83.11 ± 11.41	19.55	1.058	0.210	0.2948	0.2948
CFDA-Live	Delta	−24.65 ± 5.91	−38.29 ± 5.45	13.63	12.945	2.569	<0.0001	<0.0001
	Recovery%	69.35 ± 5.23	49.79 ± 5.33	19.56	18.498	3.671	<0.0001	<0.0001
AV^+^/PI^−^	Delta	10.26 ± 4.32	14.24 ± 5.85	−3.98	−3.867	−0.767	0.0002	0.0002
	Recovery%	193.98 ± 47.67	190.64 ± 54.87	3.34	0.325	0.064	0.7459	0.7459
PI-positive	Delta	7.86 ± 5.07	14.24 ± 3.24	−6.39	−7.491	−1.486	<0.0001	<0.0001
	Recovery%	247.25 ± 157.55	262.47 ± 65.12	−15.21	−0.629	−0.125	0.5310	0.6372
HOST-positive	Delta	−25.16 ± 4.99	−33.98 ± 5.21	8.82	8.625	1.712	<0.0001	<0.0001
	Recovery%	65.32 ± 5.94	48.53 ± 6.56	16.79	13.403	2.660	<0.0001	<0.0001
CTC-F pattern	Delta	−9.12 ± 4.51	−9.18 ± 4.76	0.06	0.066	0.013	0.9475	0.9475
	Recovery%	83.65 ± 7.21	81.56 ± 9.05	2.08	1.275	0.253	0.2054	0.3082
CTC-B pattern	Delta	4.75 ± 5.33	5.91 ± 7.90	−1.12	−0.833	−0.165	0.4074	0.6111
	Recovery%	115.19 ± 16.43	116.76 ± 22.41	−1.57	−0.399	−0.079	0.6902	0.6902
CTC-AR pattern	Delta	3.02 ± 3.00	11.67 ± 3.43	−8.65	−13.399	−2.659	<0.0001	<0.0001
	Recovery%	129.62 ± 30.77	198.2 ± 39.92	−68.89	−9.664	−1.918	<0.0001	<0.0001
PNA-Intact	Delta	−24.40 ± 5.91	−34.91 ± 4.54	10.51	9.961	1.977	<0.0001	<0.0001
	Recovery%	71.03 ± 6.41	55.82 ± 5.18	15.21	13.044	2.589	<0.0001	<0.0001
M540-High	Delta	9.28 ± 3.18	18.10 ± 5.77	−8.82	−9.454	−1.876	<0.0001	<0.0001
	Recovery%	150.82 ± 18.73	180.92 ± 33.11	−30.09	−5.593	−1.110	<0.0001	<0.0001
SCD	Delta	10.32 ± 4.98	13.69 ± 4.66	−3.36	−3.487	−0.692	0.0007	0.0009
	Recovery%	176.52 ± 48.17	176.39 ± 36.24	0.14	0.016	0.003	0.9868	0.9868
JC1-High	Delta	−22.49 ± 5.63	−25.53 ± 4.32	3.04	3.026	0.600	0.0032	0.0032
	Recovery%	62.99 ± 7.99	48.53 ± 6.69	14.46	9.810	1.947	<0.0001	<0.0001
TUNEL-Positive	Delta	6.33 ± 3.51	12.31 ± 4.16	−5.98	−7.764	−1.541	<0.0001	<0.0001
	Recovery%	177.13 ± 50.72	212.35 ± 49.57	−35.22	−3.509	−0.696	0.0007	0.0027
AO-Red	Delta	8.65 ± 3.73	12.61 ± 6.17	−3.95	−3.879	−0.769	0.0002	0.0004
	Recovery%	184.38 ± 50.62	188.96 ± 58.60	−4.58	−0.418	−0.083	0.6766	0.9868
AB-Positive	Delta	5.95 ± 4.96	8.36 ± 4.67	−2.41	−2.498	−0.496	0.0142	0.0142
	Recovery%	169.74 ± 62.75	166.85 ± 46.69	2.89	0.261	0.052	0.7945	0.9869

Delta was calculated as Frozen − Fresh. Recovery% was calculated as 100 × Frozen/Fresh. Values are presented as mean ± SD. All functional endpoints were analyzed in *n* = 50 GF and *n* = 50 PF samples, with no missing values. GF-PF indicates the difference between good and poor freezers for each metric. Between-group comparisons were performed using Welch’s *t*-test. Effect sizes are reported as Hedges g. *q*-values represent Benjamini–Hochberg false discovery rate-adjusted *p*-values. *p*- and *q*-values below 0.0001 are reported as <0.0001.

**Table 3:** Post-thaw thermoresistance of good and poor freezer samples during incubation at 37 °C.

Parameter	GF	PF	GF-PF	Welch *t*	Hedges g	*p* Value
TMOT 60 min (%)	40.88 ± 4.54	20.51 ± 3.66	20.37	–	–	–
TMOT 120 min (%)	30.24 ± 3.99	11.93 ± 2.87	18.31	–	–	–
TMOT AUC 60–120	2133.67 ± 155.66	973.47 ± 152.97	1160.20	37.589	7.460	<0.0001
PMOT 60 min (%)	28.38 ± 3.80	9.93 ± 2.17	18.45	–	–	–
PMOT 120 min (%)	18.14 ± 5.11	5.14 ± 2.11	13.00	–	–	–
PMOT AUC 60–120	1395.92 ± 207.75	452.44 ± 99.74	943.48	29.524	5.859	<0.0001

Values are presented as mean ± SD. Total motility (TMOT) and progressive motility (PMOT) were assessed after 60 and 120 min of post-thaw incubation at 37 °C in *n* = 50 GF and *n* = 50 PF samples. AUC was calculated using the trapezoidal rule: AUC = 0.5 × (value at 60 min + value at 120 min) × 60. Higher AUC values indicate greater post-thaw motility endurance. Comparisons of AUC values between GFs and PFs were performed using Welch’s *t*-test. Effect sizes are reported as Hedges g. *p*-values below 0.0001 are reported as <0.0001.

**Table 4:** Exploratory Western blot analysis of candidate cryoresistance-associated proteins in fresh pre-freeze sperm samples.

Parameter	GF	PF	GF-PF	Welch *t*	Hedges g	*p*-Value	*q*-Value
proAKAP4	0.83 ± 0.19	0.49 ± 0.10	0.34	3.220	1.980	0.027	0.075
AKAP4	1.39 ± 0.53	1.11 ± 0.46	0.28	0.803	0.494	0.453	0.453
SPAG6	0.87 ± 0.22	0.50 ± 0.06	0.37	3.299	2.029	0.038	0.075
HSP70	1.06 ± 0.34	0.91 ± 0.13	0.15	0.849	0.522	0.445	0.453
ACR	1.02 ± 0.10	1.22 ± 0.18	−0.20	−1.909	−1.174	0.119	0.191
proACR	0.76 ± 0.16	0.47 ± 0.13	0.30	2.845	1.750	0.031	0.075
proAKAP4/AKAP4 ratio	0.65 ± 0.25	0.48 ± 0.14	0.17	1.145	0.704	0.307	0.409
proACR/ACR ratio	0.75 ± 0.17	0.39 ± 0.11	0.37	3.639	2.237	0.015	0.075

Values are presented as mean ± SD. Western blot analysis was performed on a selected exploratory subset of four good freezers (GFs) and four poor freezers (PFs). The samples were selected based on sufficient sperm concentration for lysis and adequate total protein yield after extraction. Band intensities were quantified using Bio-Rad Image Lab software from non-saturated ChemiDoc images and normalized to TGX stain-free total lane protein. proAKAP4 and AKAP4 were quantified as separate AKAP4-immunoreactive bands, while proACR and ACR were quantified as separate acrosin-immunoreactive bands. Between-group comparisons were performed using Welch’s *t*-test, and effect sizes are reported as Hedges g. *q*-values represent Benjamini–Hochberg false discovery rate-adjusted *p*-values across the exploratory WB panel. Because of the small sample size, these results were interpreted as exploratory and hypothesis-generating.

## Data Availability

The datasets generated and/or analyzed during the current study are not publicly available due to participant confidentiality and institutional policy but are available from the corresponding author on reasonable request.

## Supplementary Materials

The following supporting information can be downloaded at: https://www.mdpi.com/article/10.3390/cells15161480/s1, Table S1: Fresh and frozen-thawed sperm quality in good and poor freezers; Table S2: Individual normalized Western blot densitometry values; Figure S1: Representative TGX stain-free gel used for total lane protein normalization; Figure S2: Original multiplex immunoblot for proAKAP4, AKAP4, and HSP70; Figure S3: Original multiplex immunoblot for SPAG6, proACR, and ACR.
