# Supplementary material for: Functional and Structural Determinants of Human Sperm Cryoresistance: Insights from Motility, Mitochondrial and Chromatin Integrity Analyses
Source: Cells. 2026 Aug 18;15(16):1480. doi: 10.3390/cells15161480 (PMC13510777; doi:10.3390/cells15161480)
Supplement: Supplementary file 1 [file cells-15-01480-s001.zip › cells-4425436-supplementary.pdf]

**Supplementary figures**

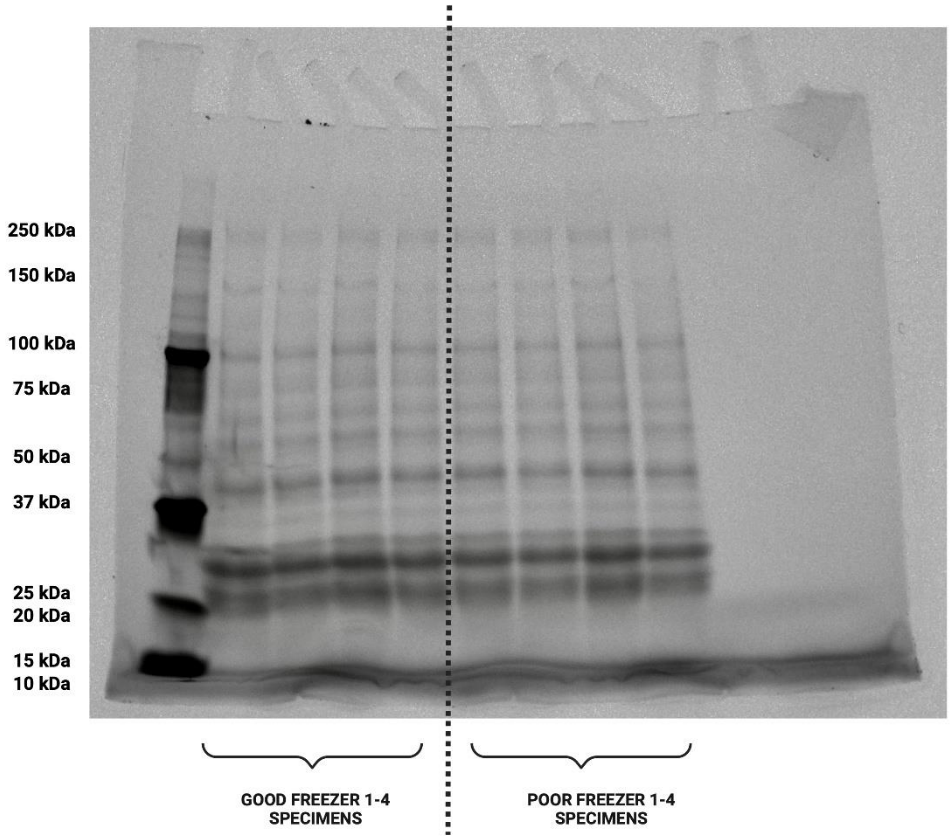


**Figure S1. Representative TGX stain-free gel used for total lane protein normalization.** Stain-free TGX gel image showing total protein loading across the Western blot samples. The total lane signal was used for normalization of target protein band intensities. The first four sample lanes correspond to good freezers and the remaining four sample lanes correspond to poor freezers.

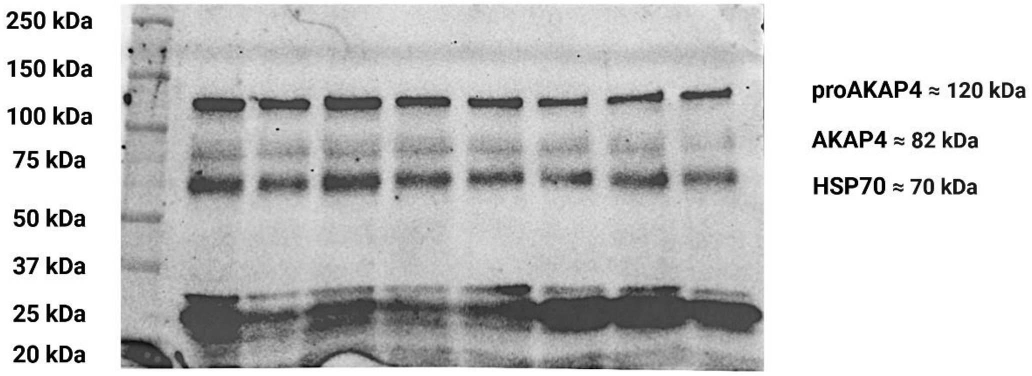


**Figure S2. Original multiplex immunoblot for proAKAP4, AKAP4, and HSP70.** Original Western blot image showing immunoreactive bands corresponding to proAKAP4 (~120 kDa), AKAP4 (~82 kDa), and HSP70 (~70 kDa) in fresh pre-freeze sperm samples. The first four sample lanes correspond to good freezers and the remaining four sample lanes correspond to poor freezers. proAKAP4 and AKAP4 were detected as separate AKAP4-immunoreactive bands using the same primary antibody and quantified according to their expected molecular weights.

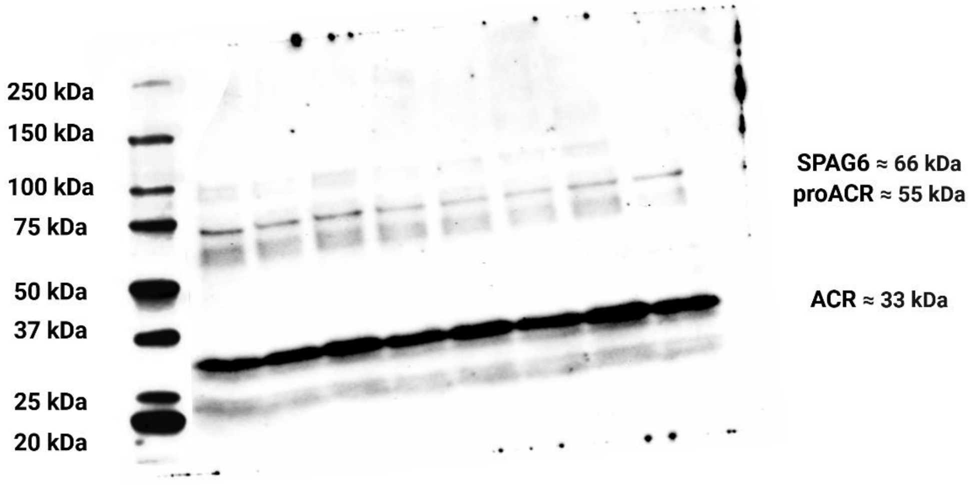


**Figure S3. Original multiplex immunoblot for SPAG6, proACR, and ACR.** Original Western blot image showing immunoreactive bands corresponding to SPAG6 (~66 kDa), proACR (~55 kDa), and ACR (~33 kDa) in fresh pre-freeze sperm samples. The first four sample lanes correspond to good freezers and the remaining four sample lanes correspond to poor freezers. proACR and ACR were detected as separate acrosin-immunoreactive bands using the same primary antibody and quantified according to their expected molecular weights.

Supplementary Table S1. Fresh and frozen-thawed sperm quality in good and poor freezers.

| Parameter | GF - Fresh | PF - Fresh | GF - Frozen | PF - Frozen |
|---|---|---|---|---|
| TMOT (%) | 72.98±5.72 | 69.04±5.65 | 48.00±5.66 | 26.30±7.83 |
| PMOT (%) | 58.56±5.87 | 54.02±6.20 | 35.56±3.75 | 15.86±4.76 |
| VAP (μm/s) | 71.04±6.32 | 69.48±6.36 | 54.37±4.57 | 40.08±8.65 |
| VSL (μm/s) | 52.54±4.49 | 49.99±4.54 | 39.56±6.65 | 27.67±5.22 |
| LIN (%) | 61.48±3.56 | 59.60±4.76 | 56.30±5.03 | 49.68±7.59 |
| ALH (μm) | 3.80±0.62 | 3.77±0.39 | 3.22±0.61 | 2.74±0.51 |
| BCF (Hz) | 25.98±5.25 | 23.21±3.75 | 22.02±3.28 | 19.02±2.15 |
| CFDA-Live (%) | 80.02±3.75 | 76.02±4.49 | 55.34±2.95 | 37.74±3.61 |
| $AV^+/PI^-$ (%) | 11.77±2.06 | 17.73±3.88 | 22.04±3.17 | 31.97±3.77 |
| PI-positive (%) | 7.36±2.39 | 9.50±2.14 | 15.22±3.82 | 23.70±2.62 |
| HOST-positive (%) | 72.30±3.06 | 65.86±3.14 | 47.16±3.74 | 31.88±3.72 |
| CTC-F pattern (%) | 55.00±3.63 | 49.04±3.76 | 45.88±3.66 | 39.86±4.15 |
| CTC-B pattern (%) | 33.06±2.82 | 38.54±3.91 | 37.86±4.81 | 44.48±6.41 |
| CTC-AR pattern (%) | 11.96±2.27 | 12.60±2.19 | 15.00±2.23 | 24.30±2.73 |
| PNA-intact (%) | 83.88±3.13 | 78.98±3.24 | 59.56±4.75 | 44.04±4.11 |
| M540-High (%) | 18.60±2.27 | 23.54±3.30 | 27.90±3.68 | 41.62±4.00 |
| JC1-High (%) | 60.32±3.28 | 49.42±3.58 | 37.84±3.78 | 23.90±3.07 |
| SCD (%) | 14.93±2.97 | 19.32±3.72 | 25.26±4.03 | 33.02±3.73 |
| TUNEL-Positive (%) | 9.22±2.44 | 11.72±2.29 | 15.48±3.22 | 24.02±3.54 |
| AO-Red (%) | 11.62±2.83 | 16.08±3.62 | 20.26±2.66 | 28.68±4.53 |
| AB-Positive (%) | 10.10±2.60 | 14.20±3.12 | 16.08±4.03 | 22.56±3.78 |

Values are presented as mean ± SD for n=50 GF and n=50 PF samples. GF, good freezers; PF, poor freezers; TMOT, total motility; PMOT, progressive motility; VAP, average path velocity; VSL, straight-line velocity; LIN, linearity; ALH, amplitude of lateral head displacement; BCF, beat-cross frequency; CFDA, carboxyfluorescein diacetate; AV+/PI-, Annexin V-positive/PI-negative spermatozoa; PI, propidium iodide; HOST, hypo-osmotic swelling test; CTC, chlortetracycline; F, non-capacitated pattern; B, capacitated pattern; AR, acrosome-reacted pattern; PNA, peanut agglutinin; M540, merocyanine; JC1, mitochondrial membrane potential marker; SCD, sperm chromatin dispersion; AO, Acridine Orange; AB, Aniline Blue.

Table S2. Individual normalized Western blot densitometry values.

| Sample | Group | proAKAP4 | AKAP4 | HSP70 | SPAG6 | proACR | ACR | proAKAP4/ AKAP4 | proACR/AC R |
|---|---|---|---|---|---|---|---|---|---|
| 1 | GF | 1.00 | 1.00 | 1.00 | 1.00 | 1.00 | 1.00 | 1.00 | |
| 2 | GF | 0.71 | 1.08 | 0.80 | 1.09 | 0.64 | 0.94 | 0.66 | |
| 3 | GF | 0.98 | 2.16 | 1.55 | 0.77 | 0.70 | 1.16 | 0.45 | |
| 4 | GF | 0.63 | 1.32 | 0.90 | 0.61 | 0.71 | 0.97 | 0.47 | |
| 1 | PF | 0.62 | 1.70 | 1.09 | 0.55 | 0.60 | 1.18 | 0.37 | |
| 2 | PF | 0.45 | 0.95 | 0.82 | 0.53 | 0.42 | 1.02 | 0.47 | |
| 3 | PF | 0.47 | 1.18 | 0.92 | 0.48 | 0.54 | 1.46 | 0.39 | |
| 4 | PF | 0.41 | 0.60 | 0.81 | 0.42 | 0.30 | 1.20 | 0.68 | |
